# Cartesian MPnRAGE for Efficient Simultaneous Multi-Contrast and Quantitative Relaxometry Imaging

**DOI:** 10.1002/mrm.70486

**Published:** 2026-07-07

**Authors:** Carly A. Allen, Kevin M. Johnson, Bernadette T. Gillick, Maren E. Schimek, Andrew L. Alexander, Steven R. Kecskemeti

**Affiliations:** 1 Department of Medical Physics, The University of Wisconsin-Madison, Madison, Wisconsin, USA; 2 Department of Radiology, The University of Wisconsin-Madison, Madison, Wisconsin, USA; 3 Department of Pediatrics, The University of Wisconsin-Madison, Madison, Wisconsin, USA; 4 Waisman Center, The University of Wisconsin-Madison, Madison, Wisconsin, USA; 5 Department of Psychiatry, The University of Wisconsin-Madison, Madison, Wisconsin, USA

**Keywords:** double inversion recovery, inversion recovery, MPRAGE, quantitative imaging, relaxometry, T1 mapping, T1-weighted imaging

## Abstract

**Purpose:**

T1-weighted images with different weighting and T1 quantification can be useful in the differentiation of both native and pathologic tissue; however, acquiring multiple images requires substantial time. A rapid and robust brain imaging sequence was developed to simultaneously provide quantitative T1 maps and multiple T1-weighted images.

**Methods:**

A novel 3D Cartesian MPnRAGE sequence was developed that collects multiple frames at different inversion times with an interleaved variable density Poisson sampling. Varying flip angles were used to enable B_1_ corrected T1 quantification from the multiple frames. Test–retest of quantitative T1 and a comparison between Cartesian MPnRAGE and reference values were performed in a phantom. Cartesian MPnRAGE was then acquired in vivo for five human participants.

**Results:**

In under 5 min, Cartesian MPnRAGE generated 10 high-quality T1-weighted images of different contrasts across the inversion recovery curve, including white matter nulled, gray matter nulled, and cerebral spinal fluid nulled frames. The 10 inversion times at varying flip angles produced accurate T1 values compared to gold standard T1 fitting, and test–retest scans resulted in low variation. In vivo scan results demonstrate the feasibility of Cartesian MPnRAGE acquisition.

**Conclusion:**

This work shows the ability of the Cartesian MPnRAGE sequence to provide accurate and efficient whole brain quantitative T1 mapping and multiple image contrasts in a reasonable acquisition time, offering an alternative acquisition strategy to existing approaches.

## 1 | Introduction

Traditional T1-weighted (T1w) images, such as MPRAGE [1], are standard in brain MRI protocols; however, there is a growing interest in acquiring additional image contrasts for improved visualization of brain structures and pathology. For example, FGATIR (Fast Gray Matter Acquisition T1 Inversion Recovery) was developed to selectively null the signal from white matter to better highlight subcortical gray matter for applications such as deep brain stimulation [2, 3]. Acquiring double inversion recovery images [4], with both CSF and white matter (WM) nulled, also provides the benefit of highlighting gray matter (GM), which may be used in clinical applications such as detecting cortical lesions in multiple sclerosis patients [5–9] or in epilepsy [10, 11]. Though these scans acquire beneficial contrasts for these clinical applications, adding these sequences in addition to the structural T1w images comes with a scan time penalty, resulting in a need for a faster way to acquire similar contrasts.

In addition to acquiring multiple contrasts, the acquisition of quantitative T1 (qT1) maps of the brain has been shown to be a valuable metric in clinical and research settings for diagnosis of disease, monitoring disease progression [12], and studying brain development [13–15] and aging [16]. Further, qT1 may enable greater specificity in diagnosis and prognosis of diseases such as multiple sclerosis, epilepsy, brain tumors, and strokes [12]. Quantitative T1 scans may also be used to improve research studies on brain development and neurological conditions such as autism, Parkinson’s disease, and dementia by allowing for comparisons between different sites and comparison between time points in large research studies [12]. However, qT1 mapping in the brain is not yet routine clinical practice, in part because adding extra scans to already lengthy protocols presents a major barrier to implementation.

Advances in qT1 imaging in the brain have begun to address this issue, with the goal of providing accurate qT1 maps in short scan times. Look-Locker [17, 18] based approaches were some of the first Cartesian qT1 mapping implemented in the brain, and many variations of the sequence have been proposed [19–23]. These methods are sensitive to B_1_ inhomogeneities and have been found to underestimate T1 [24]. Another approach to Cartesian qT1 mapping is the variable flip angle (VFA) approach [25], but this method is also sensitive to B_1_ inhomogeneities; therefore, a B_1_ map is often acquired in addition to correct for these errors [26]. A variation of the commonly implemented 3D T1-weighted (T1w) MPRAGE [1] sequence is MP2RAGE, which acquires an additional inversion time gradient echo block to create a receive bias-corrected T1w image and also a qT1 map [27]. However, the additional inversion time block in MP2RAGE often increases scan time compared to MPRAGE [27–30]. Methods have been presented to improve MP2RAGE speed [31, 32], but they remain sensitive to inversion efficiency, which has been shown to be as low as 85% in in vivo brain imaging [33]. To address the inversion efficiency bias, MP3RAGE methods add a third gradient echo inversion time block to allow inversion efficiency fitting in addition to qT1, but require a long scan time [34, 35]. Our group has developed MPnRAGE, a radial qT1 mapping sequence which also fits for inversion efficiency [36]. However, Cartesian sequences may provide different benefits than radial sequences. They have faster reconstruction times due to the utilization of the fast Fourier transform rather than a non-uniform fast Fourier transform. Cartesian sequences are similar to currently implemented clinical pulse sequences and easier to integrate with advanced reconstruction methods. Further, 3D radial sequences may require additional calibration measurements for trajectory corrections, which increase the complexity of the reconstruction. A similar method called Shuffled MPRAGE was proposed, providing multiple contrasts [37], however, it lacks qT1 mapping.

To address the need for a sequence that provides high quality T1w images, multiple inversion recovery contrasts and qT1 mapping of the brain in a modest scan time, this work aimed to develop Cartesian MPnRAGE (CaMPnRAGE) [38]. This method uses a kt-variable density Poisson [39, 40] undersampling pattern and variable flip angles with multiple gradient echo blocks acquired at different inversion times. The CaMPnRAGE sequence simultaneously provides high quality, whole brain T1w structural images, multiple inversion time contrasts, and qT1 maps in under 5 min.

## 2 | Methods

### 2.1 | PSD Development

**FIGURE 1 |:**
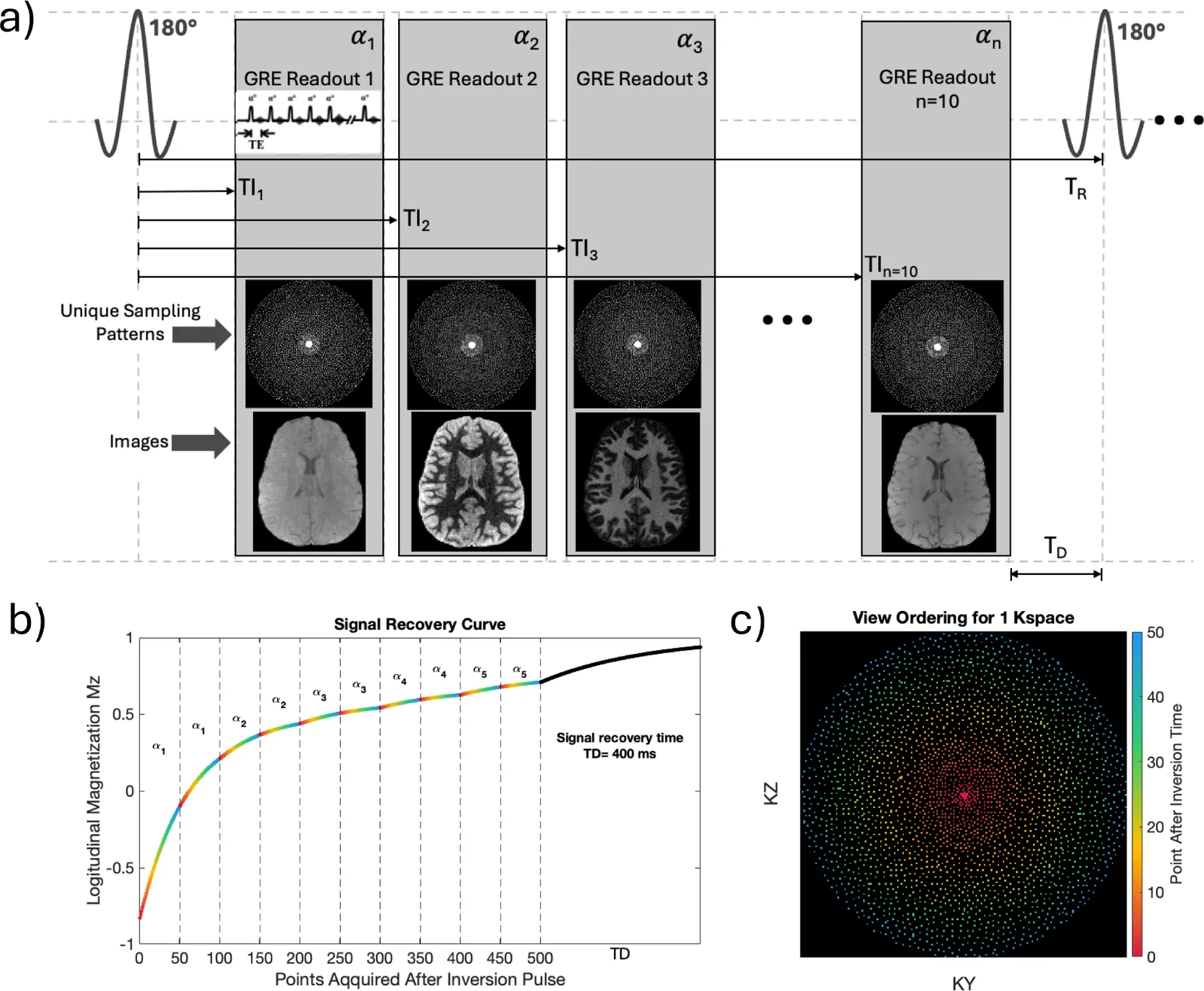
Acquisition. (a) Pulse sequence diagram of Cartesian MPnRAGE. After a magnetization prep (MP) pulse is applied, 10 gradient echo readout blocks are acquired at different inversion times, leading to unique contrast images and flip angles. Each repetition of the MP pulse adds points to a unique Poisson under sampling pattern for each frame. (b) Signal recovery curve after an MP pulse. Five-hundred points along the recovery curve are acquired, 50 points for each frame. Colors represent the point ordering shown in (c), which shows the sampling order for one frame over all views. Points closest to the inversion time are placed in the center of k-space to minimize view sharing at the lower spatial frequencies.

CaMPnRAGE follows a similar design structure to MPnRAGE, which leverages 3D radial k-space sampling following an inversion preparation pulse to acquire spatial-encoding data along the entire inversion recovery curve. In this study, a Cartesian 3D MPRAGE [1] pulse sequence was adapted to collect MPnRAGE data (Figure 1a). The gradient echo readouts after the inversion preparation pulse were partitioned into n gradient echo blocks along the inversion recovery curve. For acquisitions with a kx readout, each gradient echo block used center-out Poisson variable density ky–kz sampling to fill out k-space. The k-space sampling was pseudo-randomly varied both across the *n* inversion time blocks and all of the inversion pulses. To minimize view-sharing artifacts, points in the center of k-space were sampled as close as possible to the set inversion time of the frame, with high frequency k-space points collected further from the set inversion time, shown in Figure 1b,c. There is an inverse relationship between the number of inversion time blocks, *n*, and the number of k-space samples in each block. Higher n will result in more under sampling for each block. Based on preliminary data, 10 inversion time blocks provide a balance between unique contrasts and k-space under sampling, and results in inversion time contrasts that are roughly WM-, GM-, and CSF-nulled.

Flip angles were varied across the gradient echo blocks to enable estimates of B_1_. Gradient echo flip angles were decreased linearly from 8 to 4 degrees in steps of one degree every two gradient echo blocks so that two gradient echo blocks were obtained at each flip angle. The linear decrease in flip angle provided more regrowth toward the end of the gradient echo readout, which enabled a shorter delay time. Constant area gradient spoiling of 2π phase on each axis was applied at the end of each TR.

Poisson variable density under sampling patterns were designed using an in-house generation code to minimize T1 blurring and have unique points from the neighboring inversion time frames to reduce repeated sampling. The radius between points at the edge of k-space was varied to achieve the desired under sampling. The center of k-space was fully sampled, with the amount of sampling falling off as $\frac{1}{kr^{p}}$, with *p* = 0.3 until the radius between points reached 2.8 at the edge of k-space. Unique sampling patterns were generated for each frame as shown in Figure 1a. After each repetition of the magnetization preparation (MP) pulse, 500 total points were collected, 50 for each frame. The signal recovery curve is shown in Figure 1b. MP pulses followed by 10 gradient echo readout blocks were repeated until the sampling patterns were acquired for each frame.

### 2.2 | Acquisition Parameters

Whole brain, 1.0 mm isotropic resolution imaging was performed using a 3 T head-only scanner (MAGNUS, GE Healthcare). The acquisition was axial with the readout in the anterior–posterior (AP) direction and a field of view of 25.6 × 19.2 × 19.2 cm with 192 slices each 1 mm thick. After inversion, 500 lines of k-space were collected from 15 ms to 3109 ms in increments of TR = 6.1 ms. Inversion times for each frame were designed to be the time of the first point collected for each frame. Inversion time blocks occur in rapid succession, with a delay time of 400 ms incorporated after collecting all the blocks to allow the signal regrowth before the next MP pulse. The total time between inversion pulses is 3509 ms. Each individual inversion time frame is 14.5% sampled, with respect to the corner cut grid. The composite sampling pattern, formed by summing unique phase and partition encodes across the 10 gradient echo blocks, was greater than 94% sampled compared to the corner cut grid, resulting in a total acquisition time of 4 min and 51 s.

### 2.3 | Reconstruction and Parameter Fitting

Image reconstruction was performed using in-house python code. Images at the 10 inversion times collected from CaMPnRAGE were reconstructed with an iterative local low rank approach, minimizing the nuclear norm of 32 × 32 × 32 neighborhoods around each voxel. Our approach was similar to the one described in Reference [41], except the local low rank was performed across the 10 inversion time frames, not coils. Coil sensitivity maps were created from low resolution, zero-filled images using data from frames 4 through 10 and were utilized during the iterative reconstruction. The following equation describes the reconstruction optimization:

$$\hat{X}=arg\;\underset{x}{min}\left\{\alpha \sum_{b\in \Omega }{\left\|R_{b}X\right\|}_{*}+\|AX-Y{\|}_{F}^{2}\right\}$$

In this equation, X represents our 10 frames and α is a regularization parameter > 0, which is determined by multiplying 0.001 by the maximum singular value across all the neighborhoods on the first iteration. R is an operator that obtains the neighborhood around pixel b from each frame, b is a pixel index within our block of interest Ω, A is the SENSE [42] operator, and Y is the measured k-space data. Once the 10 contrast images were reconstructed, these images were used in a multi-pass T1 fitting procedure [43] implemented in C++ to estimate T1, B_1_, inversion efficiency and a scaling factor which includes proton density and coil sensitivities. After T1 fitting was complete, additional denoising on the 10 contrast images was performed using a global singular value thresholding algorithm, keeping the first three singular values. A brain extraction and n4 bias field correction of the images using FSL [44, 45] was performed. Whole brain WM, GM, and CSF masks were extracted from FSL fast segmentation [46] of the composite image, formed by summing the 10 complex images across all frames. Whole brain subject-specific median qT1 values of WM, GM, and CSF were averaged across each adult subject for comparison to literature values, both with and without inversion efficiency accounted for in the T1 fitting procedure. Using the acquired image contrasts, a CSF-WM nulled image similar to a double inversion recovery or FGATIR sequence [3], created by multiplying frame 2 and 5.

### 2.4 | Phantom Studies

To test the accuracy of our T1 fitting, we obtained CaMPnRAGE qT1 measurements on a caliberMRI systems mini hybrid phantom which has multiple T1 phantom vials with reference values traced to a National Institute of Standards and Technology (NIST) report [47]. Linear regression was used to compare the qT1 values of CaMPnRAGE to an inversion recovery spin echo (IR-SE) sequence. Additionally, we also performed a linear regression with qT1 values where inversion efficiency was not included as part of the fitting procedure to evaluate the importance of including inversion efficiency. We also performed a test–retest of CaMPnRAGE on the phantom over two sessions roughly 8 h apart and calculated the coefficient of variation (CoV) for each vial.

### 2.5 | Human Studies

Scans on all humans were conducted with informed consent and were in compliance with protocols approved by the UW-Madison Institutional Review Board. CaMPnRAGE was collected on four adult participants (3 female, 1 male, age 19–30) and one infant participant (female, 12 months). The gradients were derated for the infant case to reduce acoustic noise to facilitate imaging during natural sleep, resulting in a TR of 7.6 ms and a scan time of 5 min and 53 s.

## 3 | Results

**FIGURE 2 |:**
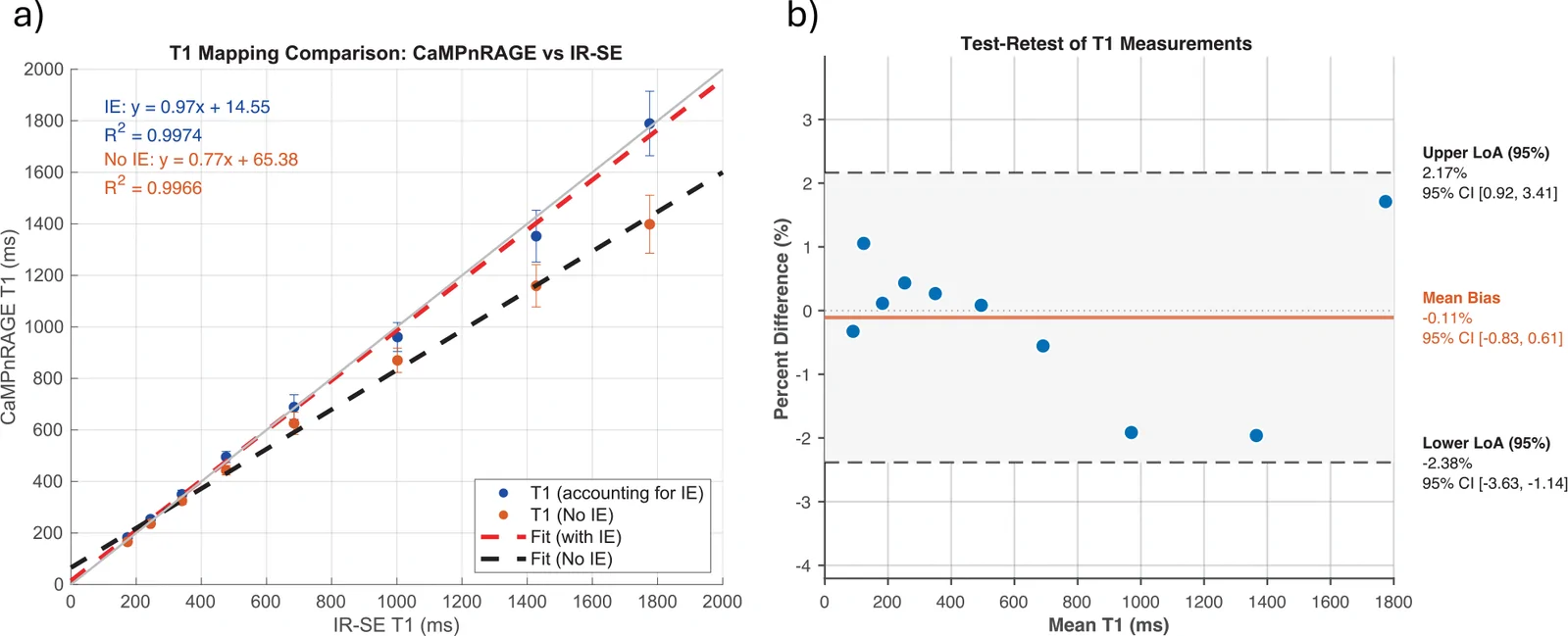
Phantom results. (a) A regression plot comparing Cartesian MPnRAGE with inversion-recovery spin echo (IR-SE). The red fit shows the regression with inversion efficiency accounted for, which has a slope of 0.97 and an intercept of 14.55 ms with an R^2^ of 0.9974. The black line shows the fit when inversion efficiency is not accounted for in the fitting procedure. In this case, the slop is 0.77 and the intercept is 65.38 ms. (b) A Bland–Altmann plot showing the differences between the test–retest T1 values in the phantom. The percent differences stay below 2%, and the mean bias is—0.11%.

Figure 2 shows the results of the phantom validation tests. In Figure 2a, the regression fit compares our CaMPnRAGE sequence with the IR-SE results. Vials with T1 values of less than 150 ms were excluded as an insufficient number of points were obtained across the recovery curve to provide accurate T1 fitting. The best fit line had a slope of 0.97 and an intercept of T1 = 14.55 ms with an R^2^ of 0.9974. The regression line that did not account for inversion efficiency in the fitting procedure has a larger bias of 65.38 ms and a slope of 0.77. In Figure 2b, the results of the test–retest for CaMPnRAGE are shown in a Bland-Altmann plot. The coefficients of variation between the tests are low, with an average of 0.62% and a standard deviation of 0.59%. A table of the coefficients of variation is included in Figure S1.

**FIGURE 3 |:**
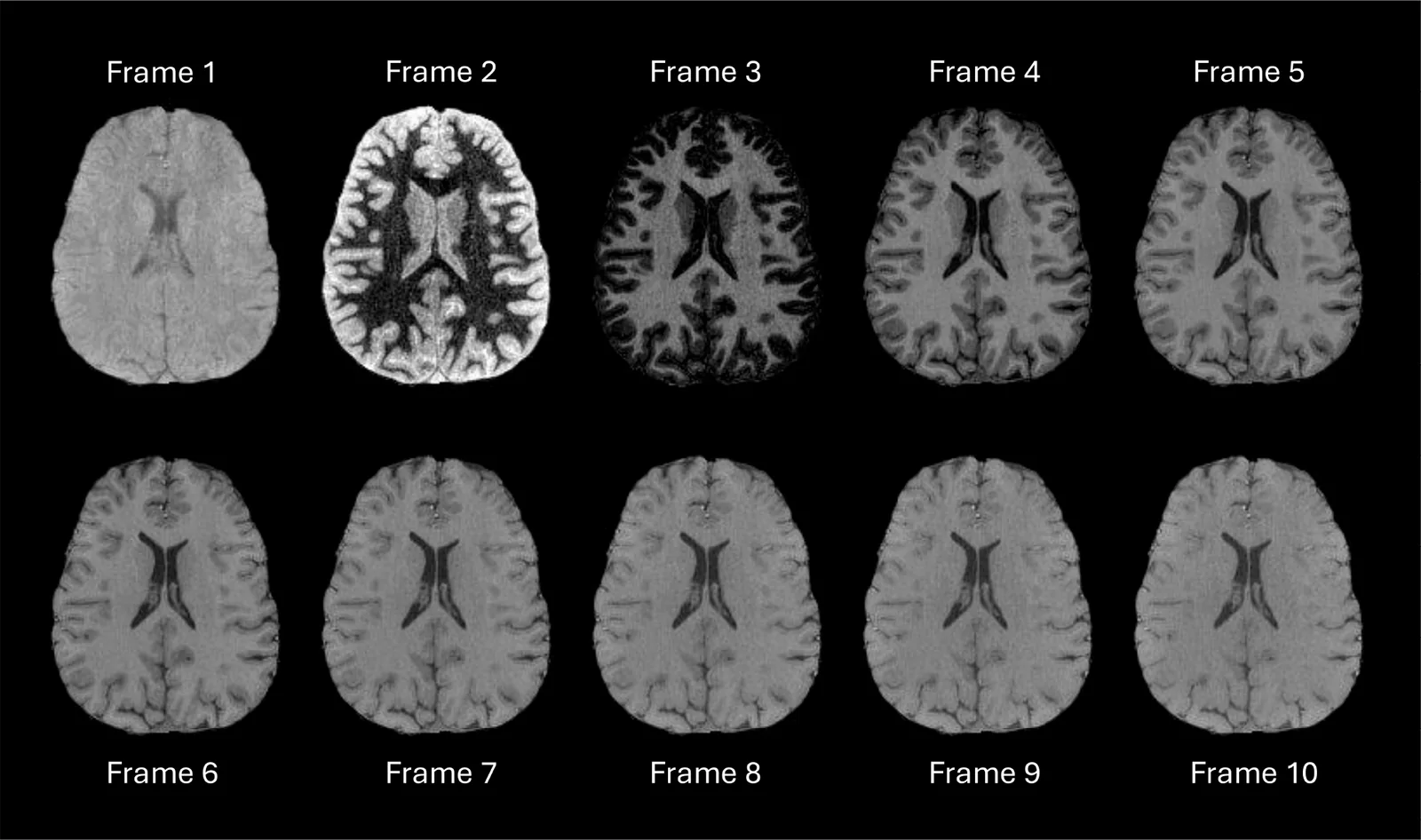
Ten T1w contrasts at the 10 different inversion times acquired in CaMPnRAGE. The signal changes more rapidly in the first half of the images. Frame 2 is WM nulled, Frame 3 is GM nulled, and Frames 3–5 are CSF nulled. Frames 4 and 5 have a T1w contrast typical to MPRAGE protocols.

For the in vivo cases, we acquired 10 different inversion time contrast images in each of the five subjects. An example of these 10 inversion time images for subject A (24 F) is shown in Figure 3, which shows that by splitting the acquisition into 10 inversion times, we acquire unique contrasts. Frame 1 occurs before the null point of white matter and looks somewhat like a proton density weighted image. Frames 2 and 3 shows roughly WM nulled and GM nulled contrast, respectively. Frames 4 and 5 are similar to traditional T1w contrast, such as an MPRAGE [3]. Frames 3–5 are CSF nulled. Frames 6–10 have roughly similar contrasts, as much of the signal has regrown and the flip angles are not high enough to provide sufficient T1-weighting.

**FIGURE 4 |:**
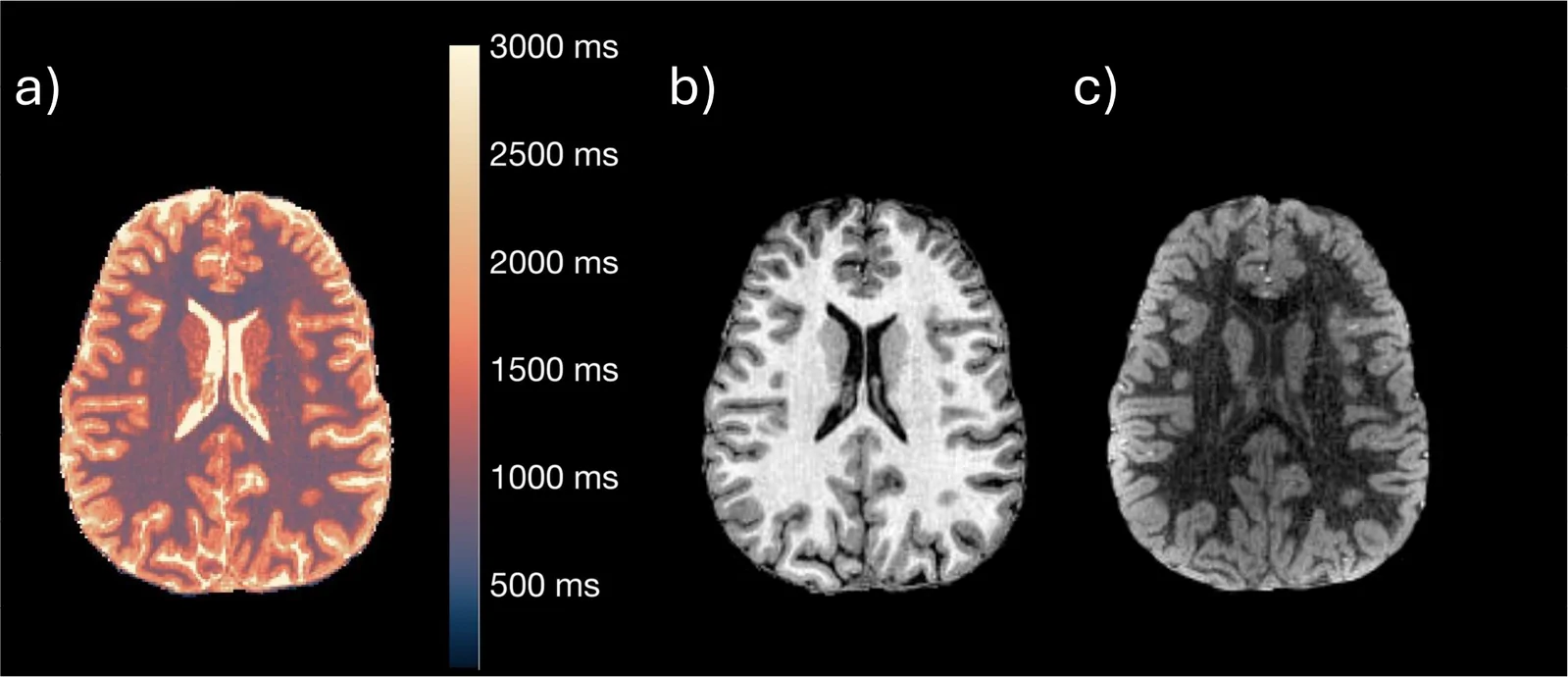
Derived Images from subject A. (a) Quantitative T1 map. (b) T1w composite image made from all frames. (c) Double inversion recovery-like image created using Frames 2 and 5 from Figure 3.

**FIGURE 5 |:**
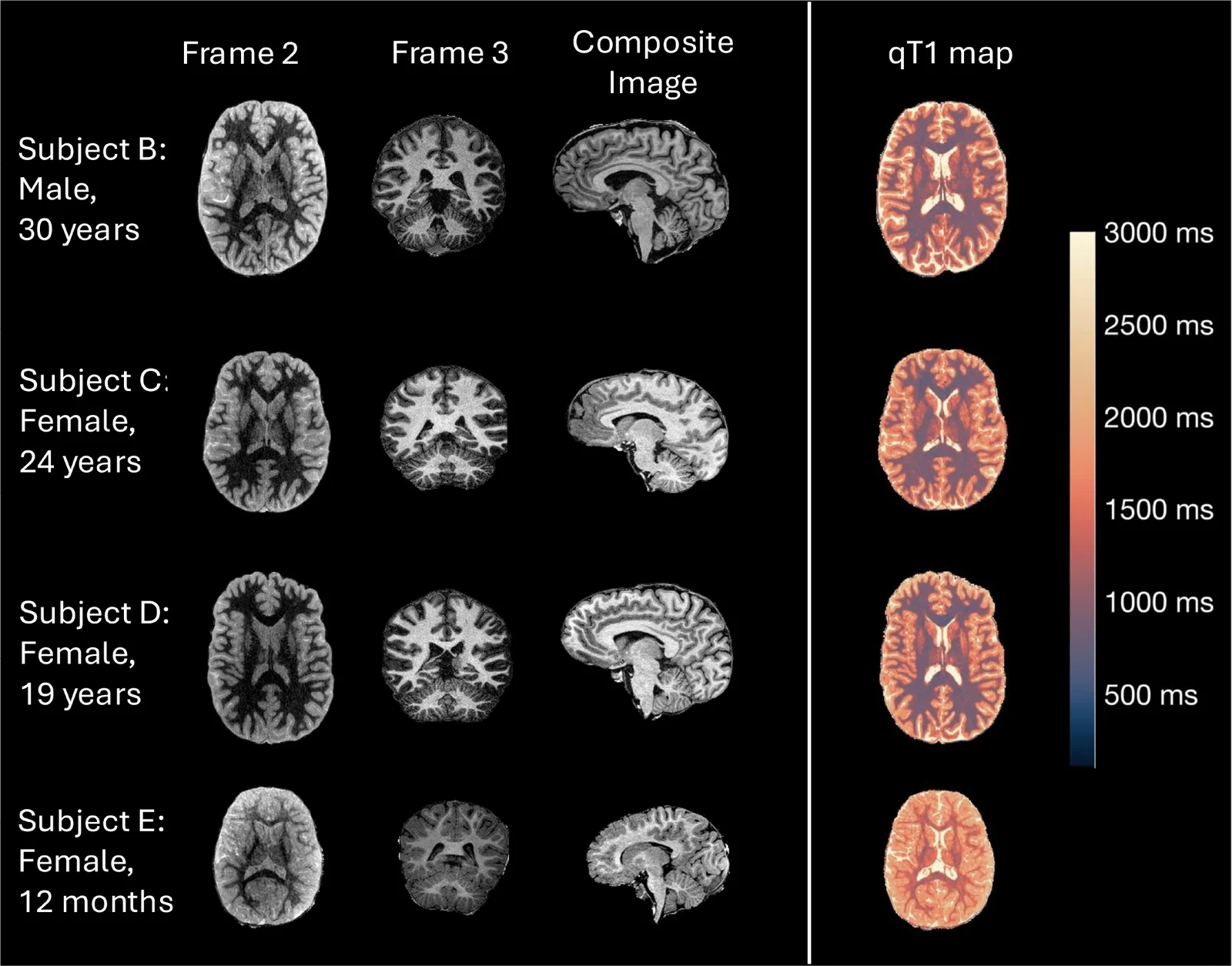
Montage of four additional subjects scanned with our protocol, including an infant (Subject E, 12 months). Three key contrasts are shown in three orientations (axial, sagittal, and coronal) as well as an axial slice of the qT1 maps for each subject.

Quantitative T1 maps are shown in Figures 4a and 5. After segmentation, masks were eroded by two voxels using FSL. For each of the four adult subjects, median qT1 values per tissue type were first computed, shown in Figure S3. The mean and standard deviation of these subject-specific median values across the four adult subjects were as follows: white matter T1: 935 ± 17 ms; gray matter T1: 1611 ± 33 ms; CSF T1: 4464 ± 192 ms. For comparison, without accounting for inversion efficiency in the T1 fitting procedure, the mean and standard deviation of the subject-specific median qT1 values were as follows: white matter T1: 719 ± 23 ms; gray matter T1: 1197 ± 10 ms; CSF T1: 2867 ± 120 ms. The other resulting images are shown in Figure 4b,c. The composite image shows a T1w contrast, while the double inversion recovery-like image nulls WM and CSF for better gray matter visualization. Figure 5 shows three additional subjects, displaying key contrasts (WM-nulled, GM-nulled, T1w) and qT1 maps in different orientations. Figure 5 also includes an infant’s scan images (Subject E), which have increased qT1 values that affect the nulling of WM and GM. Figure S2 shows all fitting maps derived from this sequence, including the scaling factor, B1, and an inversion efficiency map.

## 4 | Discussion

Our results demonstrate the feasibility of CaMPnRAGE to use Cartesian k-space sampling to generate multiple contrasts and accurate quantitative T1 maps in about the same time as commonly used MPRAGE protocols, such as the ADNI protocol [28]. In the in vivo tests, we acquire different contrasts by segmenting our acquisition into frames with different inversion times in the acquisition and reconstruction. As discussed in the introduction, the WM-nulled, GM-nulled, and CSF-nulled contrasts have been shown to have clinical utility and may be used to produce double inversion recovery-like images. This generates double inversion recovery images without additional scan time. The multiple inversion time frames also allow for qT1 fitting. Phantom results show that over the range of likely T1 values in the brain (> 150 ms), there is excellent correspondence, demonstrating the accuracy of our method. Test–retest results demonstrate high repeatability of our sequence. The bias in the regression when inversion efficiency is not accounted for demonstrates the importance of accounting for inversion efficiency to achieve accurate T1 fitting. In vivo qT1 maps in adults are also consistent with the reported range of literature values for WM, GM, and CSF qT1 [48–56].

CaMPnRAGE provides a fast alternative to other brain T1-weighted and qT1 sequences. With a scan time similar to an MPRAGE [28–30], our sequence simultaneously provides quantitative T1 images in addition to T1w images without significantly increasing total scan time which may eventually facilitate the routine acquisition of quantitative T1 in the clinical setting. CaMPnRAGE provides alternatives to other quantitative T1 sequences, such as MP2RAGE. The Poisson under sampling and view sharing allows for an increase in scanning speed. Our fitting method of fitting for B_1_, T1, inversion efficiency and a scaling factor accounts for the inversion efficiency changing throughout the brain [33], while this is not included within the MP2RAGE acquisition and fitting procedure [27]. While Shuffled MPRAGE segments an MPRAGE readout to provide multiple contrasts, Cartesian MPnRAGE provides qT1 fitting in addition, with a variable flip angle trajectory and a variable density Poisson sampling pattern rather than random sampling [35].

This work presents the feasibility of the CaMPnRAGE sequence to provide multiple image contrasts and qT1 maps. Further optimization to both reduce scan time and to improve upon the acquired image contrasts is beyond the scope of this work but may be done in the future. Flip angles, the amount of points per frame, and number of frames can all be further adjusted to change the contrasts and optimize for different ages. For example, the qT1 values of infants are different, so the inversion time parameters may be adjusted to achieve optimal WM-, GM-, and CSF-nulled frames and the traditional T1w frame. Alternatively, using the qT1 map and the pseudo-proton density map, synthetic T1w images with different weightings can be calculated to achieve nulled frames in these populations if desired [57–59]. In the future, the number of frames, n, will be explored in more detail. Here, *n* = 10 frames was chosen, as it resulted in roughly WM-, GM-, and CSF- nulled contrast frames; however, this may be adjusted to a smaller or larger number of frames. The minimum n is four for effective and accurate qT1 fitting, as we are fitting for four unknowns, T1, B1, inversion efficiency, and a scaling factor. Therefore, at minimum four independent measurements are needed for fitting. As discussed in the Methods section, there is a tradeoff between the number of points sampled per frame and the total number of frames. As the number of frames increases, the total number of unique points sampled decreases since the center of k-space is sampled for each frame to ensure each image contrast has enough low frequency information for adequate image quality. Other advanced reconstruction methods may be applied in future work to further decrease scan time, such as compressed sensing, AI methods, etc. Even from this simple local low rank reconstruction, we achieve fast scan times with multiple contrasts and accurate qT1 maps. A limitation of this method to note is that magnetization transfer (MT) effects are not accounted for with this sequence, which could affect the in vivo qT1 maps [54, 60, 61]. Previously, an in vivo comparison was performed between Fast Spin Echo — Inversion Recovery (FSE-IR) and radial MPnRAGE [36], which found similar levels of MT effects in the two methods. Because our sequence utilizes an inversion pulse and a similar method, TR, and flip angles as radial MPnRAGE, we predict comparable MT effects to both radial MPnRAGE and FSE-IR. However, future work is required to fully investigate the MT bias in CaMPnRAGE. One strength of radial MPnRAGE is that it is robust in the presence of motion. Currently, motion correction is not yet included as a part of the Cartesian MPnRAGE reconstruction. However, future work will include motion compensation, with the goal of making CaMPnRAGE comparable to radial MPnRAGE in the presence of motion. Additional future work will include expanding reconstruction techniques, further optimization of scan parameters, and reproducibility studies in more participants. Scans in infants between 0 and 24 months are currently being collected as a part of a child brain development study, with data analysis to come in the future.

## 5 | Conclusion

In this work, we present CaMPnRAGE, a novel pulse sequence that simultaneously acquires multiple image contrasts and whole brain qT1 maps in a scan time of less than 5 min. This work shows the feasibility of applying this sequence to brain protocols to acquire contrasts such as WM-, GM-, and CSF-nulled, as well as traditional T1w contrasts. We propose this method as an alternative option to T1w sequences in clinical or research brain scanning protocols, as it acquires qT1 maps in addition to T1w contrasts. We have shown through phantom scans the accuracy and repeatability of the qT1 maps and have shown the feasibility in vivo through multiple subjects. This method has the potential to reduce barriers that prevent the acquisition of quantitative relaxometry data in the clinic and during research brain scanning protocols.

## Supplementary Material

Supplementary Figures

Additional supporting information can be found online in the Supporting Information section. **Figure S1:** The coefficient of variation between the two test–retest phantom scans for eight T1 vials. **Figure S2:** All fitted maps derived from CaMPnRAGE for subject A. (a) T1 map. (b) Scaling factor. (c) B1 map. (d) Inversion efficiency map. Some WM, GM, and CSF structure can be seen in the inversion efficiency map. This is due to the dif-ferent T1 and T2 values of these tissues, as T1 and T2 relaxation occurs as the inversion pulse is applied. **Figure S3:** Median qT1 values with lower and upper quartiles in brackets for each of the four adult subjects in white matter, gray matter, and CSF. The upper bound of T1 fitting for CSF was 5000 ms. The bottom row shows the mean of the subject-specific qT1 values and standard deviation across subjects for the three tissue types.

## Data Availability

Research data are not shared.

## References

[1] Mugler JP and Brookeman JR, “Three-Dimensional Magnetization-Prepared Rapid Gradient-Echo Imaging (3D MP RAGE),” Magnetic Resonance in Medicine 15 (1990): 152–157. 2374495 10.1002/mrm.1910150117

[2] Paprad T, Yutthakasaemsan T, and Prakkamakul S, “Imaging of the Centromedian Thalamic Nucleus Using 3D Fast Gray Matter Acquisition T1 Inversion Recovery Sequence in 3T MRI in Epilepsy,” Chulalongkorn Medical Journal 70 (2026): 227–235.

[3] Sudhyadhom A, Haq IU, Foote KD, Okun MS, and Bova FJ, “A High Resolution and High Contrast MRI for Differentiation of Subcortical Structures for DBS Targeting: The Fast Gray Matter Acquisition T1 Inversion Recovery (FGATIR),” NeuroImage 47 (2009): T44–T52. 19362595 10.1016/j.neuroimage.2009.04.018

[4] Redpath TW and Smith FW, “Use of a Double Inversion Recovery Pulse Sequence to Image Selectively Grey or White Brain Matter,” British Journal of Radiology 67 (1994): 1258–1263. 7874427 10.1259/0007-1285-67-804-1258

[5] Geurts JJG, Pouwels PJW, Uitdehaag BMJ, Polman CH, Barkhof F, and Castelijns JA, “Intracortical Lesions in Multiple Sclerosis: Improved Detection With 3D Double Inversion-Recovery MR Imaging,” Radiology 236 (2005): 254–260. 15987979 10.1148/radiol.2361040450

[6] Calabrese M, De Stefano N, Atzori M, , “Detection of Cortical Inflammatory Lesions by Double Inversion Recovery Magnetic Resonance Imaging in Patients With Multiple Sclerosis,” Archives of Neurology 64 (2007): 1416. 17923625 10.1001/archneur.64.10.1416

[7] Wattjes MP, Lutterbey GG, Gieseke J, , “Double Inversion Recovery Brain Imaging at 3T: Diagnostic Value in the Detection of Multiple Sclerosis Lesions,” AJNR. American Journal of Neuroradiology 28 (2007): 54–59. 17213424 PMC8134107

[8] Granata F, Morabito R, Mormina E, , “3T Double Inversion Recovery Magnetic Resonance Imaging: Diagnostic Advantages in the Evaluation of Cortical Development Anomalies,” European Journal of Radiology 85 (2016): 906–914. 27130050 10.1016/j.ejrad.2016.02.018

[9] Jaffray A, Graf C, Rund A, , “C-DIR: Double Inversion Recovery With Controlled Artifact Suppression in Brain MRI,” AJNR. American Journal of Neuroradiology 47, no. 7 (2026): 1913–1919. 41545309 10.3174/ajnr.A9167 PMC13322368

[10] Soares BP, Porter SG, Saindane AM, Dehkharghani S, and Desai NK, “Utility of Double Inversion Recovery MRI in Paediatric Epilepsy,” British Journal of Radiology 89 (2016): 20150325. 26529229 10.1259/bjr.20150325 PMC4985945

[11] Li Q, Zhang Q, Sun H, Zhang Y, and Bai R, “Double Inversion Recovery Magnetic Resonance Imaging at 3 T: Diagnostic Value in Hippocampal Sclerosis,” Journal of Computer Assisted Tomography 35 (2011): 290–293. 21412105 10.1097/RCT.0b013e3182073c56

[12] Margaret Cheng H, Stikov N, Ghugre NR, and Wright GA, “Practical Medical Applications of Quantitative MR Relaxometry,” Journal of Magnetic Resonance Imaging 36 (2012): 805–824. 22987758 10.1002/jmri.23718

[13] Eminian S, Hajdu SD, Meuli RA, Maeder P, and Hagmann P, “Rapid High Resolution T1 Mapping as a Marker of Brain Development: Normative Ranges in Key Regions of Interest,” PLoS One 13 (2018): e0198250. 29902203 10.1371/journal.pone.0198250 PMC6002025

[14] Deoni SCL, Dean DC, O’Muircheartaigh J, Dirks H, and Jerskey BA, “Investigating White Matter Development in Infancy and Early Childhood Using Myelin Water Faction and Relaxation Time Mapping,” NeuroImage 63 (2012): 1038–1053. 22884937 10.1016/j.neuroimage.2012.07.037 PMC3711836

[15] Dean D, Shah L, DiPiero M, , “Mapping Brain Development in Infants and Young Children Using MPnRAGE T1 Relaxometry”. In: Proceedings of the 31st Annual Meeting of the International Society of Magnetic Resonance in Medicine. Abstract #4031. London, England, UK (2022).

[16] Kupeli A, Kocak M, Goktepeli M, Karavas E, and Danisan G, “Role of T1 Mapping to Evaluate Brain Aging in a Healthy Population,” Clinical Imaging 59 (2020): 56–60. 31760278 10.1016/j.clinimag.2019.09.005

[17] Look DC and Locker DR, “Nuclear Spin-Lattice Relaxation Measurements by Tone-Burst Modulation,” Physical Review Letters 20 (1968): 987–989.

[18] Look DC and Locker DR, “Time Saving in Measurement of NMR and EPR Relaxation Times,” Review of Scientific Instruments 41 (1970): 250–251.

[19] Zhang YT, Yeung HN, Carson PL, and Ellis JH, “Experimental Analysis of *T*_1_ Imaging With a Single-Scan, Multiple-Point, Inversion-Recovery Technique,” Magnetic Resonance in Medicine 25 (1992): 337–343. 1614317 10.1002/mrm.1910250212

[20] Deichmann R and Haase A, “Quantification of T1 Values by SNAPSHOT-FLASH NMR Imaging,” Journal of Magnetic Resonance 1969 96 (1992): 608–612.

[21] Blüml S, Schad LR, Stepanow B, and Lorenz WJ, “Spin-Lattice Relaxation Time Measurement by Means of a TurboFLASH Technique,” Magnetic Resonance in Medicine 30 (1993): 289–295. 8412599 10.1002/mrm.1910300304

[22] Brix G, Schad LR, Deimling M, and Lorenz WJ, “Fast and Precise T1 Imaging Using a TOMROP Sequence,” Magnetic Resonance Imaging 8 (1990): 351–356. 2392022 10.1016/0730-725x(90)90041-y

[23] Henderson E, McKinnon G, Lee TY, and Rutt BK, “A Fast 3D Look-Locker Method for Volumetric T1 Mapping,” Magnetic Resonance Imaging 17 (1999): 1163–1171. 10499678 10.1016/s0730-725x(99)00025-9

[24] Stikov N, Boudreau M, Levesque IR, Tardif CL, Barral JK, and Pike GB, “On the Accuracy of T1 Mapping: Searching for Common Ground,” Magnetic Resonance in Medicine 73 (2015): 514–522. 24578189 10.1002/mrm.25135

[25] Fram EK, Herfkens RJ, Johnson GA, , “Rapid Calculation of T1 Using Variable Flip Angle Gradient Refocused Imaging,” Magnetic Resonance Imaging 5 (1987): 201–208. 3626789 10.1016/0730-725x(87)90021-x

[26] Yarnykh VL, “Actual Flip-Angle Imaging in the Pulsed Steady State: A Method for Rapid Three-Dimensional Mapping of the Transmitted Radiofrequency Field,” Magnetic Resonance in Medicine 57 (2007): 192–200. 17191242 10.1002/mrm.21120

[27] Marques JP, Kober T, Krueger G, Van Der Zwaag W, Van De Moortele PF, and Gruetter R, “MP2RAGE, a Self Bias-Field Corrected Sequence for Improved Segmentation and T1-Mapping at High Field,” NeuroImage 49 (2010): 1271–1281. 19819338 10.1016/j.neuroimage.2009.10.002

[28] Jack CR, Bernstein MA, Fox NC, , “The Alzheimer’s Disease Neuroimaging Initiative (ADNI): MRI Methods,” Journal of Magnetic Resonance Imaging 27 (2008): 685–691. 18302232 10.1002/jmri.21049 PMC2544629

[29] Wang J, He L, Zheng H, and Lu ZL, “Optimizing the Magnetization-Prepared Rapid Gradient-Echo (MP-RAGE) Sequence,” PLoS One 9 (2014): e96899,. 24879508 10.1371/journal.pone.0096899 PMC4039442

[30] Lee EJ, Kim MG, Chung MS, Kim SO, Byun JS, and Yim Y, “Diagnosis of Intracranial Lesions Using Accelerated 3D T1 MPRAGE With Wave-CAIPI Technique: Comparison With Conventional 3D T1 MPRAGE,” Scientific Reports 12 (2022): 21930. 36536040 10.1038/s41598-022-25725-x PMC9763340

[31] Trotier AJ, Dilharreguy B, Anandra S, , “The Compressed Sensing MP2RAGE as a Surrogate to the MPRAGE for Neuroimaging at 3 T,” Investigative Radiology 57 (2022): 366–378. 35030106 10.1097/RLI.0000000000000849 PMC9390231

[32] Mussard E, Hilbert T, Forman C, Meuli R, Thiran J, and Kober T, “Accelerated MP2RAGE Imaging Using Cartesian Phyllotaxis Readout and Compressed Sensing Reconstruction,” Magnetic Resonance in Medicine 84 (2020): 1881–1894. 32176826 10.1002/mrm.28244

[33] Kingsley PB, Ogg RJ, Reddick WE, and Steen RG, “Correction of Errors Caused by Imperfect Inversion Pulses in MR Imaging Measurement of T1 Relaxation Times,” Magnetic Resonance Imaging 16 (1998): 1049–1055. 9839989 10.1016/s0730-725x(98)00112-x

[34] Hung WF, Chen PT, Chuang TC, Chang HC, and Wu MT, “High Resolution Volumetric T1 Mapping Using a Novel MP3RAGE Method [Abstract]. In: High Resolution Volumetric T1 Mapping Using a Novel MP3RAGE Method [Abstract]. Salt Lake City, Utah, USA” (2013).

[35] Olsson H, Andersen M, Kadhim M, and Helms G, “MP3RAGE: Simultaneous Mapping of *T*_1_ and B1+ in Human Brain at 7T,” Magnetic Resonance in Medicine 87 (2022): 2637–2649. 35037283 10.1002/mrm.29151

[36] Kecskemeti S, Samsonov A, Hurley SA, Dean DC, Field A, and Alexander AL, “MPnRAGE: A Technique to Simultaneously Acquire Hundreds of Differently Contrasted MPRAGE Images With Applications to Quantitative T1 Mapping,” Magnetic Resonance in Medicine 75 (2016): 1040–1053. 25885265 10.1002/mrm.25674 PMC4609219

[37] Cao P, Zhu X, Tang S, Leynes A, Jakary A, and Larson PEZ, “Shuffled Magnetization-Prepared Multicontrast Rapid Gradient-Echo Imaging,” Magnetic Resonance in Medicine 79 (2018): 62–70. 29080236 10.1002/mrm.26986 PMC5772996

[38] Allen C, Johnson K, Alexander A, and Kecskemeti S, “Cartesian MPnRAGE for Efficient Simultaneous Multi-Contrast and Quantitative Relaxometry Imaging,” in Proceedings of the 2025 International Society for Magnetic Resonance in Medicine Annual Meeting Abstract #4461 (2025). 10.1002/mrm.70486 PMC13527239 42415492

[39] Dwork N, Baron CA, Johnson EMI, O’Connor D, Pauly JM, and Larson PEZ, “Fast Variable Density Poisson-Disc Sample Generation With Directional Variation for Compressed Sensing in MRI,” Magnetic Resonance Imaging 77 (2021): 186–193. 33232767 10.1016/j.mri.2020.11.012 PMC7878411

[40] Levine E, Daniel B, Vasanawala S, Hargreaves B, and Saranathan M, “3D Cartesian MRI With Compressed Sensing and Variable View Sharing Using Complementary Poisson-Disc Sampling,” Magnetic Resonance in Medicine 77 (2017): 1774–1785. 27097596 10.1002/mrm.26254 PMC5074926

[41] . Trzasko JD and Manduca A, “Calibrationless Parallel MRI Using CLEAR,” in 2011 Conference Record of the Forty Fifth Asilomar Conference on Signals, Systems and Computers (ASILOMAR) (IEEE, 2011), 75–79.

[42] Pruessmann KP, Weiger M, Börnert P, and Boesiger P, “Advances in Sensitivity Encoding With Arbitrary k-Space Trajectories,” Magnetic Resonance in Medicine 46, no. 4 (2001): 638–651. 11590639 10.1002/mrm.1241

[43] Kecskemeti S and Alexander AL, “Three-Dimensional Motion-Corrected T1 Relaxometry With MPnRAGE,” Magnetic Resonance in Medicine 84 (2020): 2400–2411. 32301173 10.1002/mrm.28283 PMC7396302

[44] Jenkinson M, Beckmann CF, Behrens TEJ, Woolrich MW, and Smith SM, “FSL,” NeuroImage 62 (2012): 782–790. 21979382 10.1016/j.neuroimage.2011.09.015

[45] Smith SM, “Fast Robust Automated Brain Extraction,” Human Brain Mapping 17 (2002): 143–155. 12391568 10.1002/hbm.10062 PMC6871816

[46] Zhang Y, Brady M, and Smith S, “Segmentation of Brain MR Images Through a Hidden Markov Random Field Model and the Expectation-Maximization Algorithm,” IEEE Transactions on Medical Imaging 20 (2001): 45–57. 11293691 10.1109/42.906424

[47] Magnetic Resonance Measurement for MRI Biomarkers: Proton Spin Relaxation Times. (U.S. Department of Commerce National Institute of Standards and Technology Physical Measurement Laboratory, 2020), 1–6.

[48] Wansapura JP, Holland SK, Dunn RS, and Ball WS, “NMR Relaxation Times in the Human Brain at 3.0 Tesla,” Journal of Magnetic Resonance Imaging 9, no. 4 (1999): 531–538, 10.1002/(sici)1522-2586(199904)9:4<531::aid-jmri4>3.0.co;2-l. 10232510

[49] Gelman N, Ewing JR, Gorell JM, Spickler EM, and Solomon EG, “Interregional Variation of Longitudinal Relaxation Rates in Human Brain at 3.0 T: Relation to Estimated Iron and Water Contents,” Magnetic Resonance in Medicine 45 (2001): 71–79. 11146488 10.1002/1522-2594(200101)45:1<71::aid-mrm1011>3.0.co;2-2

[50] Rooney WD, Johnson G, Li X, , “Magnetic Field and Tissue Dependencies of Human Brain longitudinal ^1^H_2_O Relaxation in Vivo,” Magnetic Resonance in Medicine 57 (2007): 308–318. 17260370 10.1002/mrm.21122

[51] “mriToolbox—Parameter Database,” accessed August 25, 2025, https://www.mritoolbox.com/ParameterDatabase.html.

[52] Labadie C, Lee J, Rooney WD, , “Myelin Water Mapping by Spatially Regularized Longitudinal Relaxographic Imaging at High Magnetic Fields,” Magnetic Resonance in Medicine 71 (2014): 375–387. 23468414 10.1002/mrm.24670

[53] Weiskopf N, Suckling J, Williams G, , “Quantitative Multi-Parameter Mapping of R1, PD*, MT, and R2* at 3T: A Multi-Center Validation,” Frontiers in Neuroscience 7 (2013): 7. 23772204 10.3389/fnins.2013.00095 PMC3677134

[54] Rioux JA, Levesque IR, and Rutt BK, “Biexponential Longitudinal Relaxation in White Matter: Characterization and Impact on T_1_ Mapping With IR-FSE and MP2RAGE,” Magnetic Resonance in Medicine 75 (2016): 2265–2277. 26190230 10.1002/mrm.25729 PMC4837078

[55] Baudrexel S, Nöth U, Schüre J, and Deichmann R, “T_1_ Mapping With the Variable Flip Angle Technique: A Simple Correction for Insufficient Spoiling of Transverse Magnetization,” Magnetic Resonance in Medicine 79 (2018): 3082–3092. 29052267 10.1002/mrm.26979

[56] Berg RC, Leutritz T, Weiskopf N, and Preibisch C, “Multi-Parameter Quantitative Mapping of R1, R2*, PD, and MTsat Is Reproducible When Accelerated With Compressed SENSE,” NeuroImage 253 (2022): 119092. 35288281 10.1016/j.neuroimage.2022.119092

[57] Hagiwara A, Warntjes M, Hori M, , “SyMRI of the Brain: Rapid Quantification of Relaxation Rates and Proton Density, With Synthetic MRI, Automatic Brain Segmentation, and Myelin Measurement,” Investigative Radiology 52 (2017): 647–657. 28257339 10.1097/RLI.0000000000000365 PMC5596834

[58] Krauss W, Gunnarsson M, Nilsson M, and Thunberg P, “Conventional and Synthetic MRI in Multiple Sclerosis: A Comparative Study,” European Radiology 28 (2018): 1692–1700. 29134354 10.1007/s00330-017-5100-9 PMC5834550

[59] Hwang K and Fujita S, “Synthetic MR: Physical Principles, Clinical Implementation, and New Developments,” Medical Physics 49 (2022): 4861–4874. 35535442 10.1002/mp.15686

[60] Mossahebi P, Yarnykh VL, and Samsonov A, “Analysis and Correction of Biases in Cross-Relaxation MRI due to Biexponential Longitudinal Relaxation,” Magnetic Resonance in Medicine 71 (2014): 830–838. 23440870 10.1002/mrm.24677 PMC3674218

[61] Tsialios P, Thrippleton M, Glatz A, and Pernet C, “Evaluation of MRI Sequences for Quantitative T1 Brain Mapping,” Journal of Physics Conference Series 931 (2017): 012038.

