# Supplementary material for: Cartesian MPnRAGE for Efficient Simultaneous Multi-Contrast and Quantitative Relaxometry Imaging
Source: Magn Reson Med. Author manuscript; Available in PMC 2026 Sep 10. (PMC13527239; doi:10.1002/mrm.70486)
Supplement: Supplementary file 1 [file NIHMS2205482-supplement-Supplementary_Figures.pdf]

Figure S1: The coefficient of variation between the two test-retest phantom scans for eight T1 vials.

Test-Retest Results	
Region	Coefficient of Variation (%)
1	0.08
2	0.31
3	0.19
4	0.06
5	0.39
6	1.35
7	1.39
8	1.21

Figure S2: All fitted maps derived from CaMPnRAGE for subject A. a) T1 map. b) Scaling factor. c) B1 map. d) Inversion efficiency map. Some WM, GM, and CSF structure can be seen in the inversion efficiency map. This is due to the different T1 and T2 values of these tissues, as T1 and T2 relaxation occurs as the inversion pulse is applied.

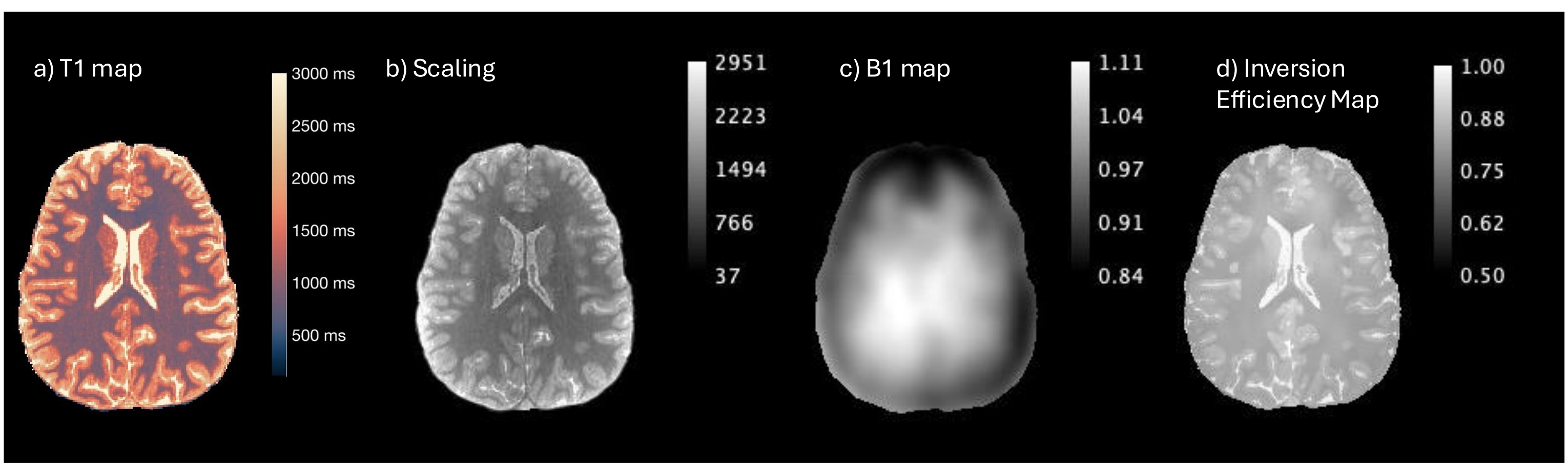

Figure S3: Median qT1 values with lower and upper quartiles in brackets for each of the four adult subjects in white matter, gray matter, and CSF. The upper bound of T1 fitting for CSF was 5000 ms. The bottom row shows the mean of the subject-specific qT1 values and standard deviation across subjects for the three tissue types.

	**Intrasubject Median qT1 [25th, 75th percentile] (ms)**		
	White Matter	Gray Matter	CSF
Subject A	942 [892, 996]	1620 [1468, 1782]	4776 [3477, 5000]
Subject B	955 [900, 1012]	1556 [1415, 1714]	4446 [3464, 5000]
Subject C	934 [883, 986]	1647 [1496, 1808]	4369 [3268, 5000]
Subject D	910 [860, 961]	1620 [1467, 1791]	4264 [3188, 5000]
Group Mean ± SD	935 ± 16	1611 ± 33	4464 ± 192
